# Optimization of alkali-treated poplar fiber saccharification using metal ions and surfactants

**DOI:** 10.1080/21655979.2020.1857576

**Published:** 2020-12-22

**Authors:** Fan Feng, Baiquan Zeng, Shilin Ouyang, Yanan zhong, Jienan Chen

**Affiliations:** a College of Life Science and Technology, Central South University of Forestry & Technology, Changsha, China; b Ministry of Forestry Bioethanol Research Center, Changsha, China; c Hunan Engineering Research Center for Woody Biomass Conversion, Changsha, China

**Keywords:** Alkali treatment, saccharification, response surface, metal ion, surfactant

## Abstract

In this study, contrary to untreated poplar fiber, processing of alkali-treated poplar fiber was optimized for the enzymatic saccharification. Considering reducing sugar content as the evaluation index, pH, temperature, time, amount of enzyme, and rotational speed of shaker were standardized to optimize the sugar production by enzymatic hydrolysis. Using response surface methodology, the optimum technological condition of enzymatic hydrolysis was found to be utilizing 43 mg cellulase at 46 °C for 50 h. At this, the sugar conversion amount of NaOH or H_2_O_2_-NaOH pretreated poplar was 164.62 mg/g and 218.82 mg/g respectively. It was a corresponding increase of 446.73% or 626.75% than that of poplar fiber without a pretreatment. At a low concentration, metal ions and surfactants promoted the conversion of reducing sugar. Especially, 0.01 g/L Mn^2+^ and 0.50 g/L hexadecyl trimethyl ammonium bromide (CTAB) produced the best effect and increased the conversion rate of reducing sugar by 23.62% and 21.44% respectively. Also, the effect of the combination of metal ions and surfactants was better than that of a single accelerator. By improving the enzymatic process, these findings could enhance the utilization of poplar fiber for the production of reducing sugar.

## GRAPHICAL ABSTRACT

**Figure:**
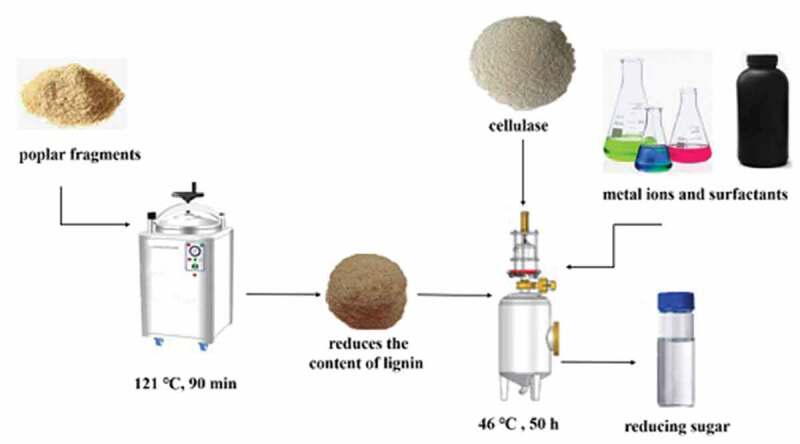

## 1. Introduction

The overexploitation of natural resources, rapidly depleting mineral energy, and the massive combustion of fossil fuels are the foremost reasons for air pollutants and the Greenhouse Effect [1]. However, by increasing the output of biomass energy, considerable consumption of fossil fuels can be counterbalanced to resolve the challenges of energy shortage, and environmental degradation [2]. Lignocellulose, a rapid growth and a wide range of sources, is an inexhaustible natural resource that can be utilized as a good raw material for the production of material and energy [3]. Poplar, rich in lignin fiber, is a kind of fast-growing tree with countless applications in papermaking, biofuel, and other industries [4].

**Figure 1.**
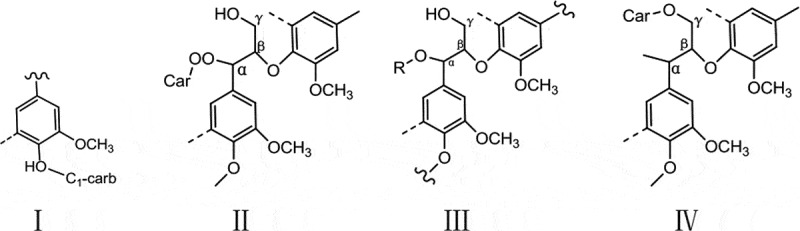
Types of lignin sugar complexes linkages

The fermentation of biomass energy such as lignocellulose is one of the hot topics at present. The crystalline zone, high degree of polymerization, surface void structure of cellulose, and the close cross-linking structure of cellulose, hemicellulose, and lignin all make the lignin fiber difficult to biodegrade. Therefore, the premise of efficient hydrolysis to produce sugar is to use efficient pretreatment technology to destroy the natural structure of lignin, remove the ingenuity of lignin and reduce the degree of polymerization of cellulose and hemicellulose. Different pretreatment methods have different enzymolysis and fermentation effects. The pretreatment technologies of lignocellulosic materials mainly include physical method, chemical method, biological method, and combined method (such as high-temperature mechanical grinding method, alkali-mechanical grinding method, direct steam explosion method, SO_2_-steam explosion method, NH_3_-steam explosion method, H_2_O_2_-steam explosion method, and CO_2_-steam explosion method) [5–8]. Therefore, it is essential to pretreat poplar and the chemical method of pretreatment is widely used. In this method, pretreatment with dilute acid solution significantly reduces the hemicellulose content in the lignin fiber but the separation of lignin is disappointing [9]. On the contrary, the pretreatment with dilute alkali solution markedly reduces the content of lignin by destroying the structure of the Lignin sugar complex (Figure 1), producing components rich in cellulose and hemicellulose [10]. Moreover, it induces the expansion of wood fibers exposing increased contact area for the enzyme cellulase. Also, hydrogen peroxide-based oxidation decomposes lignin in lignin fiber [11], reduces cleanliness, and improves the efficiency of enzymatic hydrolysis and saccharification.

Most cellulase preparations are products of microbial fermentation, such as *Trichoderma reesei, Aspergillus Niger*, and so on [12]. Importantly, metal ions are the vital nutrients required for microbial growth and cellulase production [9]. Also, metal ions function as the enzyme activators to improve the enzymatic efficiency [13]. During the enzymatic hydrolysis of fiber, cellulose is absorbed by lignin [14], which reduces the enzymatic efficiency and consequently lowers the yield of reducing sugar.

Deying [15] studied the effects of 10 kinds of metal ions on the activity of cellulase produced by the greenwood enzyme, and found that some metals have the effect of improvement on it. Chao [16]studied the effects of various chemical additives on cellulase a ctivity, but they did not do experimental research on the comparison of the promotion effects of these two kinds of additives and the deep mechanism of promoting enzymatic hydrolysis, but only made preliminary speculation, and none of them played a role in the process of the enzymatic hydrolysis and saccharification of lignocellulose. Therefore, in this study, using the alkali based delignification of poplar wood, the effects of different concentrations of metal ions and surfactants were investigated on the enzymatic production of sugar. These results may help in achieving superior enzymatic hydrolysis for sugar production and can be used as a reference for industrial and research purposes.

## 2. Materials and methods

### 2.1. Materials and instruments

The poplar planting base was in Hanshou County, China. Plant logs were naturally air-dried and crushed into 20-mesh poplar sawdust for dry storage. Cellulase was purchased from Cangzhou Xiasheng enzyme Biotechnology Co., Ltd. And the main instruments are shown in Table 1.

**Table 1.** Main instrument

Name	Type	Country
Electronic balance	AL204	America
Miniature plant grinder	FZ102	China
Automatic high pressure steam sterilizer	DAIHANLABTECH Co	Japan
Electronic universal furnace	1 KW	China
Desktop high speed centrifuge	H/T18MM	China
Electric blast drying box	WGL-230B	China
Muffle furnace	SX2-4-10 T	China
Tabletop thermostat oscillator	IS-RDD3	China
Ultraviolet visible spectrophotometer	UV-8100B	China
Precision digital display acidity meter	PHS-3B	China

### 2.2. Poplar pretreatment

Precisely 10 g poplar fragments, transferred into a conical bottle, were incubated with 4% sodium hydroxide solution for 90 min in a high-pressure steam sterilizer at 121 °C. Likewise, 10 g poplar fragments, transferred into a conical bottle, were incubated with a mixed solution of 4% sodium hydroxide and hydrogen peroxide for 90 min in a high-pressure steam sterilizer at 121 °C. The pretreated poplar was washed to neutral with pure water and then dried and set aside.

### 2.3. Enzymatic hydrolysis and fermentation of poplar fiber

Precisely 1 g poplar sawdust, transferred into a 50 ml conical bottle, was treated with 30 mg cellulase for hydrolysis at pH 4.8 and 50°C with a substrate liquid to material ratio 30:1 mL/g for 48 h for hydrolysis and fermentation.

### 2.4. Effect of additives on the enzymatic hydrolysis and fermentation of poplar fiber

Different metal ions and surfactants were added to the enzymatic hydrolysis fermentation system(Tables 2 and 3), and under the optimal enzymatic hydrolysis conditions, the content of reducing sugar was determined after the fermentation.

**Table 2.** Different metal ion addition concentrations

Metal ion concentration (g/L)					
Na^+^	0.001	0.005	0.01	0.015	0.02
K^+^	0.001	0.005	0.01	0.015	0.02
Mn^2+^	0.001	0.005	0.01	0.015	0.02
Mg^2+^	0.01	0.03	0.05	0.07	0.09
Ca^2+^	0.01	0.03	0.05	0.07	0.09
Fe^2+^	0.001	0.01	0.03	0.05	0.07
Cu^2+^	0.09	0.12	0.15	0.18	0.21

**Table 3.** Different surfactant concentration

Surfactant concentration (g/L, ml/L)					
CTAB(S)	0.2	0.3	0.4	0.5	0.6
Citric acid (S)	0.1	0.3	0.5	0.7	0.9
Tween-80 (L)	0.1	0.5	0.9	1.3	1.7
Glycerol (L)	0.5	1.0	1.5	2.0	2.5

### 2.5. Analytical methods

#### 2.5.1. Characterization method

The content of cellulose was determined by the nitric acid-ethanol method. The content of hemicellulose was determined by hydrochloric acid hydrolysis. The content of lignin was determined by the concentrated sulfuric acid method. At the same time, it draws lessons from the (NREL) detection method of the National Renewable Energy Laboratory of the United States. The surface morphology of poplar was observed by using a scanning electron microscope. To measure water content, 1 g poplar was heat-dried in an oven at 105 °C and weighed every four hours until the mass reached a constant weight [17].

$$\omega (\%)=\frac{M0-M}{M0}\times 100$$

where w(%) is the water content, *M_0_* is the quality of poplar before drying (g), and *M* is the final weight of poplar after drying (g).

#### 2.5.2. Glucose standard curve

Glucose was dried to a constant weight in an oven at 105 °C. Then, a standard solution of glucose was prepared by dissolving 0.1 g of glucose in water, and volume was fixed to 100 mL. Using different dilutions, 3 mL DNS reagent was added and mixed evenly to allow the reaction in a boiling water bath for 10 minutes. This was cooled rapidly to room temperature and water was added to fix the volume to 25 mL. The absorbance was measured at 540 nm using an ultraviolet spectrophotometer. Using the absorbance value (*OD_540_*) as the ordinate and the glucose concentration (mg/mL) as the abscissa, the standard curve was obtained. Applying the regression equation: y = 1.3544x-0.0231, the regression coefficient R^2^ = 0.9992 indicated a good linear relationship.

#### 2.5.3. Determination of the enzyme activity

1 g solid cellulase powder was used for hydrolyze 50 mg filter paper at pH 4.8, 50 °C for 1 h. The amount of enzyme required for hydrolysis of filter paper to form 1 μmol glucose per hour is considered one unit of enzyme activity (U). Using the filter paper enzyme activity (FPA) method, the cellulase activity was determined to be 1500 U/g [18].

#### 2.5.4. Determination of reducing sugar

The fermented liquid was centrifuged for 10 min at 8000 r. The retrieved supernatant was diluted several times, and using the DNS colorimetric method and glucose standard curve, reducing sugar content was determined as described previously [19].

$$Y=\frac{C\times V}{M0\times (1-\omega )}$$

where *Y* is the conversion amount of reducing sugar (mg/g), *C* is the concentration of enzymatic hydrolyzate (mg/mL), *V* is the volume of enzymatic hydrolyzate (mL), *M_0_* is the undried quality of poplar (g), and *ω* is water content.

*Plackett-Burman design experiment and response surface experiment to achieve optimal enzymatic hydrolysis*

Considering the single factor results, the Plackett-Burman design of six factors, such as enzyme addition, liquid to material ratio, time, temperature, pH, and shaker speed, was carried out using the software Design expert 8.0.6. The influential factors and levels of the design experiment are shown in Table 4. Based on the results of the experiment designed by Plackett-Burman, considering the conversion amount of reducing sugar as the response value, the response surface analysis of three factors was performed at three levels.

**Table 4.** Factors and levels of Plackett-Burman design

Number	Factors	Low	High
A	Enzyme addition	30	40
B	Material-liquid ratio	10	20
C	Time	40	48
D	Temperature	40	50
E	pH	4.6	4.8
F	Shaking speed	150	200
G	-	-	-
H	-	-	-
J	-	-	-
K	-	-	-
L	-	-	-

## 3. Results and analysis

### 3.1. Single-factor experimental analysis

#### 3.1.1. Effect of temperature on the production of reducing sugar

Temperature is a significant factor that affects the hydrolysis rate of cellulase. Like most enzymes, cellulase enzyme is also a protein. Therefore, non-optimal temperature conditions inhibit cellulase activity affecting the efficiency of enzymatic hydrolysis which in turn reduces the saccharification [20,21].

**Figure 2.**
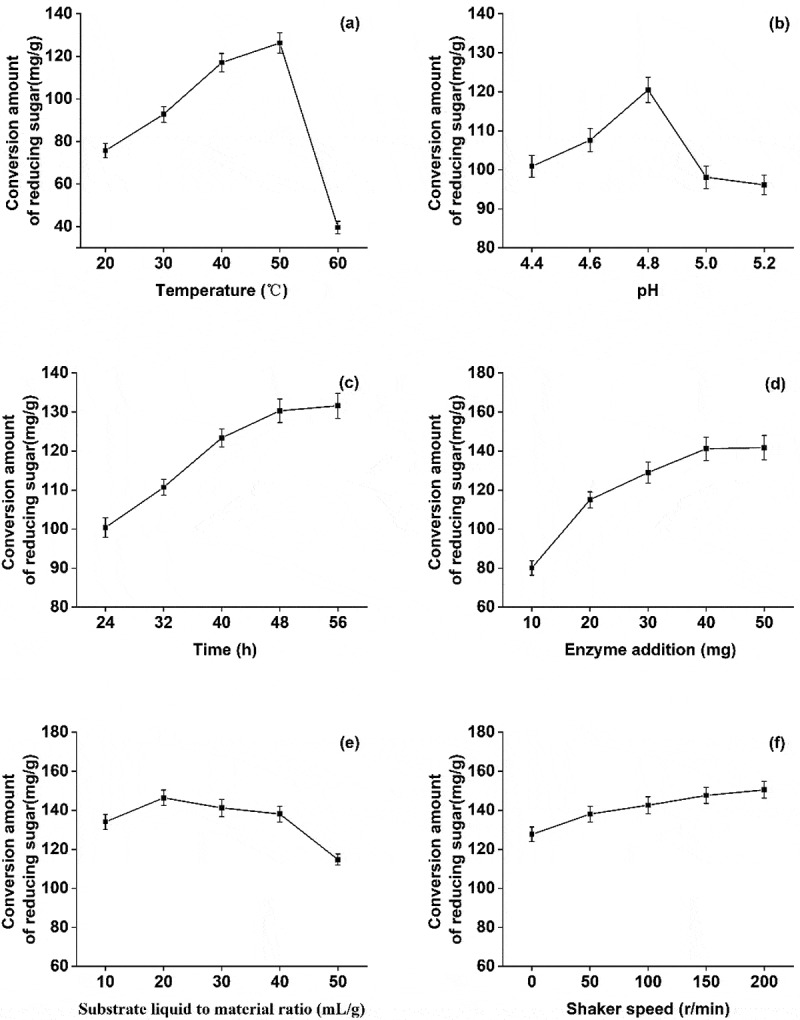
Effects of different factors on the yield of reducing sugar

Under test conditions, 30 mg cellulase was used for hydrolysis at pH 4.8 with a substrate liquid to material ratio 30:1 mL/g for 48 h. The reducing sugar content increased between 20–50 °C and then decreased sharply between 50–60 °C(Figure 2(a)). This suggested that the increase of temperature up to 50 °C boosts the cellulase activity, improves enzymatic hydrolysis, and yields of reducing sugar. However, the temperature above 50 °C inhibited the enzyme activity and was not conducive for enzymatic hydrolysis and saccharification. Therefore, 50 °C was selected as the optimum temperature for the enzymatic hydrolysis.

(a)Effect of temperature on the yield of reducing Sugar (b)Effect of pH on the yield of reducing Sugar (c)Effect of time on the yield of reducing Sugar (d)Effect of enzyme addition on the yield of reducing Sugar (e)Effect of substrate-liquid-material ratio on the yield of reducing Sugar (f)Effect of shaking speed of shaker on the yield of reducing sugar

#### 3.1.2. Effect of pH on the production of reducing sugar

Over acidic or alkali can destroy salt bonds or hydrolyze ester bonds, so under acidic or alkaline conditions cause protein denaturation and inactivation [22], and the main component of cellulase is the protein. Therefore, pH has a significant effect on the enzymatic saccharification. Studies have shown that cellulase performs better in weak acidic conditions [22,23]. Therefore, it is important to examine the effect of pH on enzymatic hydrolysis.

Like earlier, under the test conditions, 30 mg cellulase was used for hydrolysis at pH 4.8 with a substrate liquid to material ratio 30:1 mL/g for 48 h. At a pH range of 4.4–4.8, the yield of reducing sugar positively correlated with an increase of pH, and the amount of reducing sugar reached to maximum at pH 4.8. After that, the increase of pH led to a decrease in the yield of reducing sugar(Figure 2(b)). This could be due to the inactivation of enzyme cellulase that reduced the efficiency of enzymatic hydrolysis.

#### 3.1.3. Effect of time on the production of reducing sugar

In general, a longer hydrolysis time increases the yield of reducing sugar. However, a prolonged enzymatic hydrolysis causes greater loss to the instrument which reduces the efficiency and increases the cost.

Similarly, 30 mg cellulase was utilized for hydrolysis at pH 4.8, 50 °C with a substrate liquid to material ratio of 30:1 mL/g. During 24–48 hours, the yield of reducing sugar increased significantly. However, an overextension of enzymatic hydrolysis to 60 h decreased the yield of reducing sugar(Figure 2(c)). This could be due to the accumulation of by-products which hinders the production of reducing sugar [24]. Therefore, considering the cost, 48 h was selected as the optimum enzymatic hydrolysis time.

#### 3.1.4. Effect of enzyme addition on the production of reducing sugar

Though increasing the cellulase concentration can improve the efficiency of enzymatic hydrolysis, it would not be cost-effective [25]. Therefore, to find the optimal cellulase concentration, enzymatic hydrolysis was carried out at pH 4.8 with a substrate liquid to material ratio 30:1 mL/g for 48 h. For the range of 10–40 mg enzyme, the yield of reducing sugar improved with the increasing concentration of enzyme. However, beyond this range, increasing the cellulase amount did not significantly improve the yield(Figure 2(d)). On the contrary, it decelerated the production of reducing sugar, and cellulase at 50 mg concentration showed no further change in production. Due to this, and given that the cost of enzymatic hydrolysis, 40 mg was selected as the optimal amount of cellulase.

#### 3.1.5. Effect of substrate liquid to material ratio on the production of reducing sugar

Substrate liquid to material ratio has a significant effect on the yield of reducing sugar. A low liquid to material ratio is not favorable for cellulase action due to the reduced contact surface. However, a high liquid to material ratio increases the enzyme contact with poplar wood and improves the efficiency of enzymatic hydrolysis.

As earlier, 40 mg cellulase was used for hydrolysis at pH 4.8 and 50 °C for 48 h. The yield of reducing sugar increased with an increase in the liquid to material ratio from 10: 1 to 20: 1, and reached the maximum at 20: 1. However, between 20:1 and 50:1, the yield of reducing sugar decreased with an increase of material to liquid ratio(Figure 2(e)). The initial increase improved the efficiency of enzymatic hydrolysis by facilitating the full contact between the enzyme solution and poplar fiber. But, for the large liquid to material ratio, the dissolution of impurities also increased [26]. Based on these results, the optimal liquid to material ratio of 20:1 was selected for the enzymatic hydrolysis.

#### 3.1.6. Effect of shaker speed on the production of reducing sugar

As shown in Figure 2(f), an increase in the rotational speed of the shaker improved the efficiency of enzymatic hydrolysis, and the yield of reducing sugar increased gradually.

As previously, 40 mg cellulase was used for hydrolysis at pH 4.8, 50 °C with a liquid to material ratio of 20: 1 for 48 h. At a rotational speed 0–200 r/min, the yield of reducing sugar was positively correlated with the increase of rotational speed. At a higher rotational speed, the contact area between the substrate and the enzyme increases which in turn facilitates the enzymatic hydrolysis. Hence, the shaker speed 200 r/min was selected as the optimal speed.

### 3.2. Results and analysis of Plackett-Burman design experiment and response surface experiment to achieve optimal enzymatic hydrolysis

The response value of the production of reducing sugar was analyzed. As presented in Tables 5 and 6, the three factors that showed a significant influence on the response value for the production of reducing sugar were: D (temperature), C (time), and A (enzyme addition). Table 4 showed all of them had a P-value <0.05. Therefore, these three important factors were selected for the Box-Behnken experiment. Based on the results designed by Plackett-Burman, the three factors, enzyme addition, time and temperature showed a great influence on sugar production by cellulase hydrolysis. The amount of added enzyme, time, and temperature were taken as independent variables to optimize the enzyme hydrolysis parameters. The factors and levels of the design experiment are presented in Tables 7 and 8. The experimental results were analyzed and fitted by Design expert 8.0.6 software. The regression equation is as follows:

$$Y=146.64+14.56A+10.40B-47.45C-4.35AB-1.97AC-7.69BC-3.74A^{2}-4.51B^{2}-55.81C^{2}.$$

**Table 5.** Plackett-Burman design test factors and response values

	A	B	C	D	E	F	Value(R)
1	30	-1	40	40	4.6	150	94.7281
2	40	-1	48	50	4.8	150	157.062
3	30	1	48	40	4.8	200	125.417
4	30	-1	40	50	4.6	200	125.402
5	40	-1	48	50	4.6	200	156.077
6	30	1	48	50	4.6	150	144.998
7	40	1	40	40	4.6	200	127.285
8	40	1	40	50	4.8	200	138.094
9	30	-1	48	40	4.8	200	106.101
10	40	-1	40	40	4.8	150	100.047
11	30	1	40	50	4.8	150	123.291
12	40	1	48	40	4.6	150	125.789

**Table 6.** ANOVA for selected factorial model Analysis of variance table

Source	Sum of Squares	df	Mean Square	F Value	p-value Prob > F	
Model	4133.93	6	688.99	11.99	0.0077	significant
A- enzyme addition	593.85	1	593.85	10.33	0.0236	
B- substrate liquid to material ratio	172.20	1	172.20	3.00	0.1440	
C- time	946.90	1	946.90	16.48	0.0097	
D- temperature	2284.10	1	2284.10	39.75	0.0015	
E-pH	49.07	1	49.07	0.85	0.3978	
F- shaker speed	87.81	1	87.81	1.53	0.2713	
Residual	287.34	5	57.47			
Cor Total	4421.27	11

**Table 7.** Box-Benhnken test factors and their levels

Influence factor	Level		
	−1	0	1
A-Enzyme addition (mg)	30	40	50
C-Time (h)	40	48	56
C-Temperature (°C)	40	50	60

**Table 8.** Box-Benhnken test factors and response values

Number	A	B	C	Value(R)
1	0	−1	1	37.777
2	0	1	1	38.589
3	1	0	−1	149.721
4	0	0	0	146.079
5	0	0	0	150.493
6	0	−1	−1	118.679
7	−1	−1	0	106.097
8	0	0	0	147.634
9	1	0	1	52.253
10	1	1	0	161.978
11	0	1	−1	150.233
12	−1	0	−1	117.973
13	0	0	0	146.373
14	0	0	0	142.629
15	−1	1	0	140.219
16	1	−1	0	145.251
17	−1	0	1	28.398

Table 9 shows that A, B, C, and C^2^ had significant effects on the conversion of reducing sugar (p < 0.01). Through the analysis of variance, the R^2^ of the model is 0.9976, the adjusted determination coefficient R^2^_adj_ is 0.9946, and the coefficient of variation C.V. % is 2.94. These indicate that the model credibility and the degree of fitting are good. Furthermore, the model fidelity 325.62 and p < 0.0001 verifies the significance of the model. The ‘Lack of Fit F-value’ of 2.06 and p > 0.05 implies the Lack of Fit is not significant relative to the pure error and the regression model with the real value has a good fitting degree.

**Table 9.** ANOVA for Response Surface Quadratic Model Analysis of variance table [Partial sum of squares – Type III]

Source	Sum of Squares	df	Mean Square	F Value	p-value Prob > F		
Model	34,463.79	9	3829.31	325.62	< 0.0001	significant	
A- Enzyme addition	1697.00	1	1697.00	144.30	< 0.0001		
B- Time	865.59	1	865.59	73.60	< 0.0001		
C- Temperature	18,010.98	1	18,010.98	1531.54	< 0.0001		
AB	75.65	1	75.65	6.43	0.0389		
AC	15.57	1	15.57	1.32	0.2876		
BC	236.27	1	236.27	20.09	0.0029		
A2	59.03	1	59.03	5.02	0.0600		
B2	85.68	1	85.68	7.29	0.0307		
C2	13,115.26	1	13,115.26	1115.24	< 0.0001		
Residual	82.32	7	11.76				
Lack of Fit	50.01	3	16.67	2.06	0.2477	not significant	
Pure Error	32.31	4	8.08				
Cor Total	34,546.11	16					

#### 3.2.1. Interaction analysis

**Figure 3.**
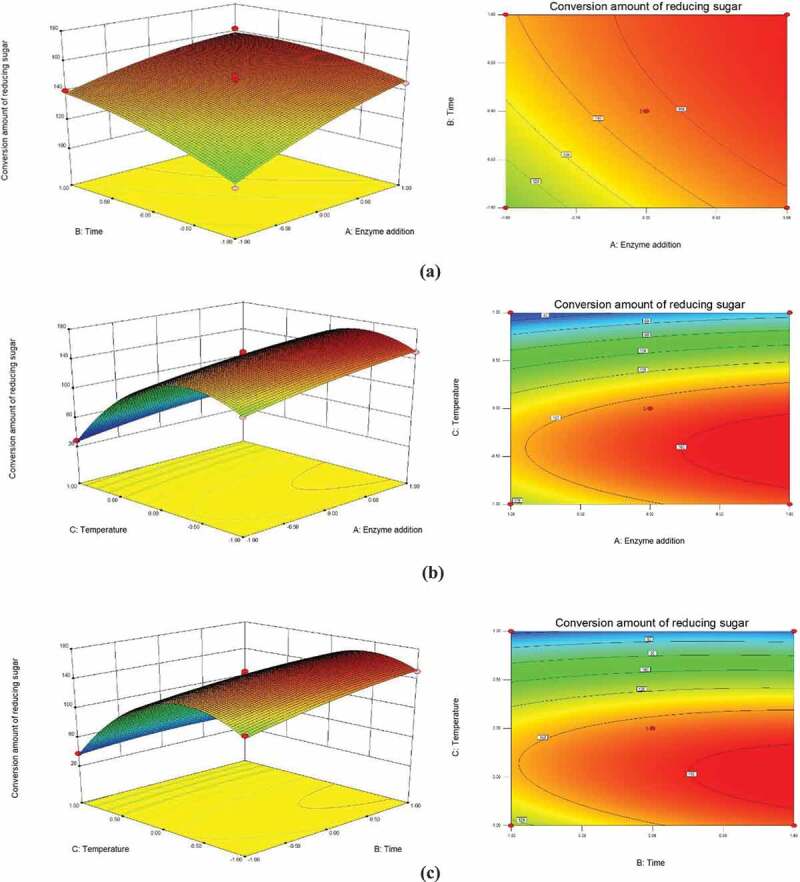
Response surface of the effect of different factors on the conversion of reducing sugar

As shown in Figure 3, the interaction of enzyme dosage-time and temperature-time has a certain effect on the conversion amount of reducing sugar. In Figure 3(a), the three-dimensional response surface is steep, and the conversion amount of reducing sugar increases with the increase of enzyme addition and hydrolysis time. Also, the contour refers to a significant interaction between enzyme addition and hydrolysis time. It can be visualized from the density of the contour map in Figure 3(b) and the slope of the three-dimensional response surface map that with an increase of pH, the production of reducing sugar first increases to the maximum and then decreases. The reduced production could be due to the inactivation of cellulase caused by the higher temperatures which in turn reduces the enzymatic efficiency. The contour map shows that the interaction between enzyme addition and temperature is not significant. Since the range of these two variables is smaller, a change in the interaction of a larger range of variables is not observed. The density of the contour shown in Figure 3(c) indicates that there is a significant interaction between enzymatic hydrolysis time and temperature.

(a) Response surface diagram and contour map of conversion amount of reducing sugar of enzyme addition and time (b) Response surface diagram and contour map of conversion amount of reducing sugar of enzyme addition and temperature (c) Response surface diagram and contour map of conversion amount of reducing sugar of time and temperature

Using the mathematical model, the best technological conditions for enzymatic saccharification were predicted as following: using 43.09 mg cellulase at 46.02 °C for 50 h should yield 163.81 mg/g of reducing sugar.

#### 3.2.2. Verification of regression mode

To verify the aforesaid model and practical operation, 43 mg cellulase was used for hydrolysis at 46 °C for 50 h. Three parallel experiments were carried out. It was found that that the conversion amount of reducing sugar was 163.91 mg/g, 165.42 mg/g, and 164.53 mg/g, respectively. Surprisingly, the final yields were significantly close to the theoretical prediction, indicating an effective model.

#### 3.2.3. Comparison of sugar production based on pretreatment methods

**Figure 4.**
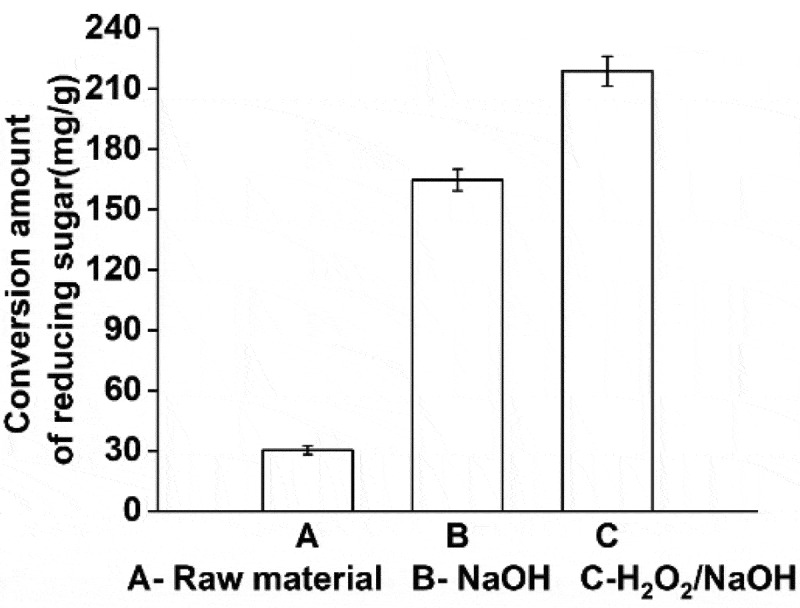
Comparison of reducing sugar production by different pretreatment methods

The poplar without pretreatment, NaOH treatment, and H_2_O_2_-NaOH treatment were fermented by enzymolysis using the same optimal conditions. As shown in Figure 4, compared to no pretreatment, poplar fiber treated with NaOH and H_2_O_2_-NaOH showed better enzymatic hydrolysis, and the yield of reducing sugar increased by 446.73% and 626.75% respectively.

### 3.3. Effect of metal ions on the conversion of reducing sugar

**Figure 5.**
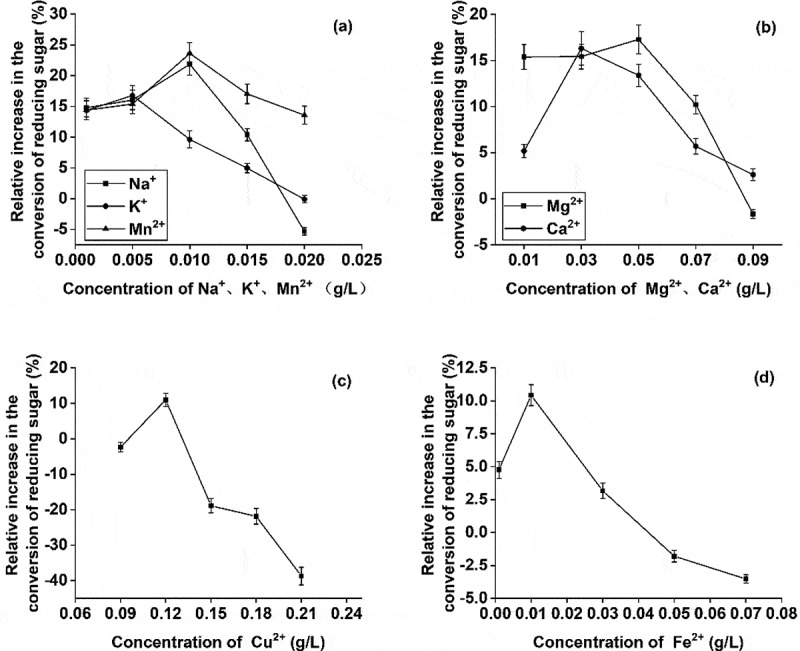
Effect of different metal ions on the conversion of reducing sugar

Various concentrations of Na^+^, Cu^2+^, Fe^2+^, K ^+^, Mn^2+^, Mg^2+^, and Ca^2+^ were added to the enzymatic fermentation system. At the end of the reaction, the production of reducing sugar was compared with a control blank system, without any metal ions. The results showed that different ions had distinct effects on enzymatic hydrolysis. Primarily, most of the metal ions with an increase in concentration at first promoted and then inhibited the production of reducing sugar (Figure 5). It seemed that cellulase activity initially increased at lower ion concentration but then decreased at higher ion concentration [27]. Mn^2+^ and Na^+^, in low concentrations, showed the greatest effect on the conversion of reducing sugar. At 0.01 g/L, the conversion amount of reducing sugar increased by 23.62% and 21.85%, respectively. Mn^2+^, a transition metal cation, belongs to the cellulase active site and promotes substrate binding to the enzyme [28]. Mg^2+^, Ca^2+^, K^+^ showed a secondary effect on the conversion of reducing sugar. Mg^2+^, at 0.05 g/L, showed the greatest effect on the yield of reducing sugar and increased it by 17.28%. However, a significant inhibitory effect was observed at a higher concentration of Mg^2+^ in the reaction system(Figure 5(a,b)). The heavy metal ions induce protein denaturation and therefore Cu^2+^ and Fe^2+^ either showed a little effect or inhibited the production of reducing sugar. At higher concentrations, these can also produce toxic effects to inactivate the cellulase. As shown in Figure 5(c,d), the inhibitory effect turned stronger with an increase in metal ion concentration.

### 3.4. Effect of surfactant on the conversion of reducing sugar

**Figure 6.**
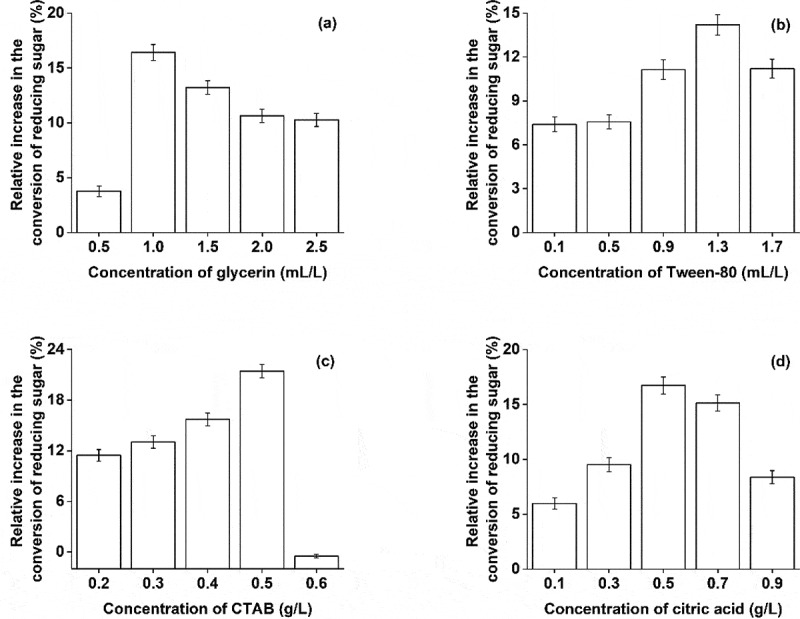
Effect of different surfactants on the conversion of reducing sugar

Surfactants, by enhancing the lignin absorption to cellulase, improve the efficiency of enzymatic hydrolysis [29]. Different concentrations of citric acid, glycerin, hexadecyl trimethyl ammonium bromide (CTAB), and Tween-80 were tested for enzymatic hydrolysis and the content of reducing sugar was determined. As shown in Figure 6, in the set range, the surfactant promoted the conversion of reducing sugar at low concentration. Among them, the effect of CTAB was strongest which increased the yield of reducing sugar by 21.44%. The promoting effect of citric acid, glycerol, and Tween-80 were second to CTAB. The surfactant reduces the surface tension between the lignin fiber and local solution which increases the dispersion of enzyme cellulase improving enzymatic hydrolysis [30]. However, with the further increase in concentration, the promoting effect of Tween-80 and CTAB changed into the inhibitory effect. It could be that at a higher concentration surfactants began to compete with the cellulase for the adsorption site, which hindered the enzymatic hydrolysis.

(a) Effect of Glycerol on the conversion of reducing Sugar (b) Effect of Tween-80 on the conversion of reducing Sugar (c) Effect of CTAB on the conversion of reducing Sugar (d) Effect of citric acid on the conversion of reducing sugar

### 3.5. The combined effect of metal ions and surfactants on the conversion of reducing sugar

**Figure 7.**
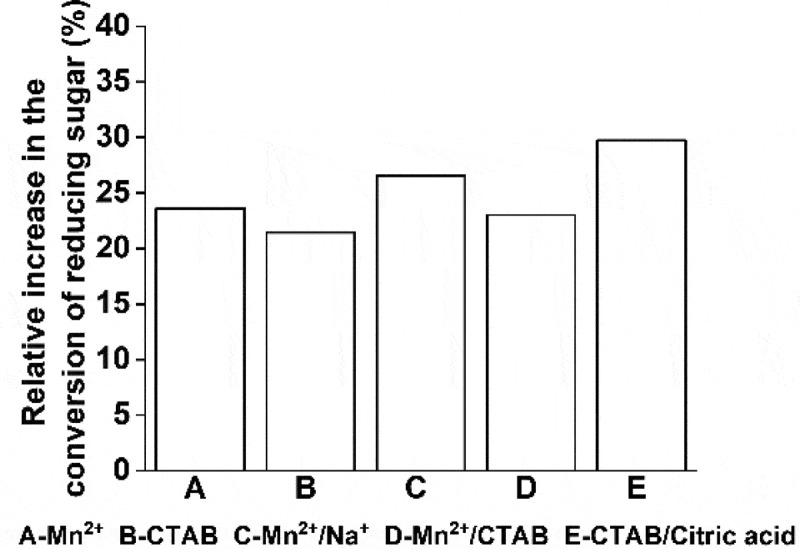
Effect of the mixture of metal ions and surfactants on reducing sugar

Based on the results presented in Figures 4 and 5, the effects of metal ion-metal ion, metal ion-surfactant, and surfactant-surfactant on the conversion of reducing sugar were investigated. Three groups of experiments, related to Mn^2+^/Na^+^, Mn^2+^/CTAB, and CTAB/citric acid, were carried out. The results show that the combined effect of the mixture of metal ions, metal ions along with surfactants, and the mixture of surfactants is better than using a single accelerator (Figure 7). However, the promoting effect of the combination of metal ions with surfactants was poor and behaved similarly to Mn^2+^ alone.

### 3.6. Determination of components in Poplar and characterization analysis

**Figure 8.**
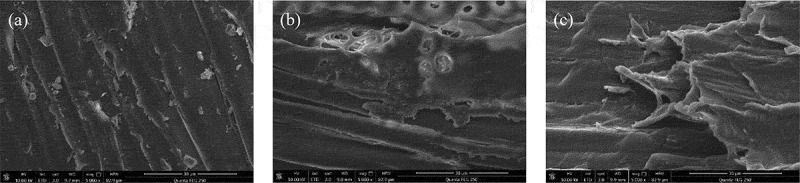
Scanning electron microscope images of poplar with different pretreatment methods

Table 10 shows that pretreatment can significantly reduce the content of lignin, expose cellulose, and participate more in the process of enzymatic hydrolysis and saccharification.Scanning electron microscopy (SEM) showed significant differences in the surface morphology of poplar treated with different methods (Figure 8). The surface structure of poplar fibers without a pretreatment looks flat and dense. Also, the fibers are arranged in an orderly manner. However, the surface of poplar fiber treated with NaOH is rough with broken holes. These features could have increased the surface area of poplar and thereby improved the degradation of hemicellulose and lignin. The surface structure of poplar treated with H_2_O_2_-NaOH was seriously damaged and turned loose and wrinkled. It appears that peroxide oxidized the chemical bonds between cellulose, hemicellulose, and lignin to produce the larger voids increasing the surface area remarkably.

**Table 10.** Effect of Pretreatment on Poplar components (%)

Pretreatment method	Cellulose	Hemicellulose	Lignin
Raw material	46.91	23.35	24.78
NaOH	64.60	14.89	19.28
H_2_O_2_- NaOH	65.50	16.40	17.32

(a)Raw material (b) NaOH pretreated poplar (c) H_2_O_2_-NaOH pretreated poplar

## 4. Discussion

The further utilization of wood fiber is an effective way to solve the problem of energy, and researchers have done a lot of exploration. In order to improve the utilization efficiency, the current trend is mainly focused on the pretreatment method, while the acid treatment method is used more [31,32], but the effect is general. In contrast, sodium hydroxide can degrade lignin in raw materials and improve the adsorption capacity of cellulase. Although hydrogen peroxide can not degrade cellulose components, it can destroy related structural bonds to enhance the effect of pretreatment. All these are beneficial to improve the effect of poplar saccharification. Due to the poor effect of direct enzymatic hydrolysis of sugar, the researchers considered adding additives in the process of cellulase hydrolysis to increase sugar production. Junying [24] added xylanase in the process of poplar enzymatic hydrolysis to make the sugar yield reach 160.15 mg/g. And Na [33] added β-glucosidase to poplar wood enzymatic hydrolysis, which made the reducing sugar yield reach 43.11 g/L. In contrast, it was found that different metal ions and surfactants had different effects on the yield of reducing sugar, which could be increased by up to 23.62%. After reaching the equilibrium concentration of adsorption, it showed inhibitory effect. And the results of the compound experiment further show that the combination of two metal ions or two kinds of surfactants is more beneficial to improve the sugar yield than the single use of two kinds of metal ions or two kinds of surfactants. Nonetheless, at present, there are few studies on the combination of metal ions and the combination of metal ions and surfactants on poplar enzymatic hydrolysis to produce sugar, which needs to be further studied. These findings are valuable for the proper utilization of poplar fiber into reducing sugar.

## 5. Conclusion

Using response surface methodology, the optimum technological condition of enzymatic hydrolysis 43 mg cellulase at 46 °C for 50 h was obtained. Enzymatic hydrolysis of NaOH or H_2_O_2_-NaOH pretreated poplar wood produced 164.62 mg/g and 218.82 mg/g of reducing sugars respectively. An effective method to improve the enzymatic saccharification of poplar was put forward. The results showed that in the range of low concentration, all metal ions and surfactants promoted the yield of reducing sugar with different intensities. Particularly, 0.01 g/L Mn^2 +^ and 0.50 g/L CTAB. produced the strongest promoting effect while Cu^2+^ and Fe^2+^ had an inhibitory effect.

## 6. Future prospective

In this study, the enzymatic saccharification efficiency of poplar was significantly improved, and it was proved that trace metal ions and surfactants could be used as potential additives to promote poplar saccharification. This study is still in the preliminary exploration stage and can not reach the level of industrial production. In the future, the efficiency of poplar saccharification can be further studied through the interaction between metal ions and surfactants.

## Highlights

- Improve the efficiency of saccharification and enzymatic hydrolysis of poplar wood by combined pretreatment.
- The optimum conditions of enzymatic hydrolysis of poplar by cellulase were optimized.
- Metal ions and surfactants were added to promote enzymatic hydrolysis so as to improve the yield of sugar.
- The effect of the interaction between metal ions and surfactants on the sugar production of poplar was discussed.
